# Reflections on Afro-descendant origin and the outcome of dengue fever cases in Colombia

**Published:** 2017-06-30

**Authors:** Aura Caterine Rengifo, María Alexandra Durán, Yamileth Ortíz, Jorge Martín Rodriguez, Martha Lucía Ospina

**Affiliations:** 1 Grupo de Morfología Celular. Instituto Nacional de Salud, Bogotá, Colombia.; 2 Red Nacional de laboratorios. Instituto Nacional de Salud. Bogotá, Colombia.; 3 Subdirección de Investigación, Científica y Tecnológica. Instituto nacional de Salud. Bogotá, Colombia.; 4 Director de Investigación en Salud Pública. Instituto Nacional de Salud. Bogotá, Colombia.; 5 Directora del Instituto Nacional de Salud de Colombia. Bogotá, Colombia.

Dear editors, cordial greetings

As a group of researchers and professionals signing this letter to the editors, respectfully, we want to make a series of observations and reflections regarding the article "Afro-Colombian ethnicity, a paradoxical protective factor against dengue" ^1^, published in your prestigious journal.

We considered that the article in question lightly concludes that the Afro-Colombian population present a lower risk of complications compared to the non-Afro-Colombian population. This situation, besides of not being completely based on data and facts that it describes and analyzes, is an assertion that can be a source of deeply disorientation for the Colombian health services, upon a time these conclusions get disseminated, and it can also mistakenly indicate to Afro-Colombian people that the risk of complications is lower in the presence of a virus causing a dengue disease.

In our point of view, several of the article's affirmations can be object of xenophobic and exclusive evaluations towards the Afro-Colombian populations, mostly perceived when the authors conclude that in zones or neighborhoods with high number of Afro-Colombian there is a greater incidence of dengue among this population. The authors did not show data or measurements of this condition to identify whether (or not) Afro-descendants were infected at the site of evaluation and whether (or not) they developed the disease, so that these findings should have been assessed more rigorously before making this kind of affirmations and "risky" conclusion.

Although there are characteristics of Afro-descendants that may give to them less susceptibility to severe dengue manifestations, it is already known about the multifactorial pathogenesis of this disease; in which, variables such as environmental factors ^2^, Age ^3^, gender ^4^, different blood types AB versus types A, O and B ^5^, allele polymorphisms of human leukocyte antigens ^6^, and factors associated with leakage Vascular...among others, play an important role.

The conditions related to host genetics and environmental factors might predispose to severe forms of the disease, but the important thing is to bear in mind that most of them have been the result of conclusions obtained by studies of association. In addition, recent meta-analysis studies on factors that may influence dengue's manifestations indicate that malnutrition has an inverse association with dengue shock syndrome and dengue hemorrhagic fever ^7^ ^,^ ^8^. We might then, paradoxically, think that "malnutrition offers a kind of protection against the development of the severe form of the disease". Therefore, being a person of black race does not mean that he/she is protected against some forms of dengue, neither it indicates that it is a risk factor for other ethnic groups ^9^.

 It is also possible that the article is failing to measure or has bias in misclassification, regarding to the auto-recognition for each patient's race ^10^. A dichotomous classification as Afro-Colombian or non-Afro-Colombian can generate measurement errors; particularly, considering that an Afro descendant patient (mulatto, zambo, mestizo, and others) can be classified as non-Afro-Colombian. Additionally, genetics of their predecessors makes it much vulnerable to possible complications of external origin infectious events. It is possible that of the 402/431 cases of severe dengue cases with complications in non-Afro-Colombians reported by the authors, several of them have genetic elements and traits of Afro-Colombian population, generating a measurement error in which it would be needed to perform Genetic and biological measurements that were not considered.

On the other hand, it is confusing the fact that when comparing with the conclusions obtained by Rojas et al. ^1^ in relation to the epidemiological reports made by the National Institute of Health (NIH) in 2013, it differs substantially. For example, the NIH reported that the most prevalent provincial o territories (in order of magnitude) with cases of classical dengue and severe dengue are Tolima, Valle, Santander, Norte de Santander and Cundinamarca. In these territories/provinces, (with the exemption of Valle) there are low black race population ^11^.

Although the authors state that there is no risk of a possible ecological fallacy, it is obvious that most conclusions were obtained with data analysis of population-level records, which exceed individual conclusions ^10^. To avoid this type of situation, multi-level or multi-step method validations are required to ensure consistency between the two measurement levels.

Finally, it should be considered that dengue is a complex disease in which mechanisms and severe forms of infection have not been fully elucidated, requiring robust and ethical studies to establish new theories about viral pathogenesis, as to avoid making wrong assumptions for the individuals under the study.
