# Supplementary material for: Reflections on Afro-descendant origin and the outcome of dengue fever cases in Colombia
Source: Colomb Med (Cali). 2017 Jun 30;48(2):98–100. (PMC5625562)
Supplement: Translation

# Translation: Reflexiones sobre el origen de los afrodescendientes y los casos de dengue en Colombia

Estimados editores

El grupo de investigadores y profesionales firmantes de esta carta al editor, queremos realizar una serie de observaciones-reflexiones muy respetuosas, con relación al artículo "Afro-Colombian ethnicity, a paradoxical protective factor against dengue" **** ^1^, publicado en su prestigiosa revista. 

Consideramos, que el artículo en mención, concluye de forma ligera que la población afrocolombiana presentó menor riesgo de complicaciones, comparado con la población no afrocolombiana; esta situación, además de no estar completamente basada en los datos y hechos que describe y analiza, es una afirmación que puede ser profundamente desorientadora para los servicios de salud, quienes una vez diseminadas estas conclusiones, pueden de forma errada indicarle a los usuarios afrocolombianos que el riesgo de complicaciones es menor ante la presencia de una virosis causada por el Dengue. 

Varias de las afirmaciones del artículo, pueden ser objeto de apreciaciones xenofóbicas y excluyentes hacia las poblaciones afro-colombianas, tal y como se percibe cuando los autores concluyen que en las zonas o barrios con alta población afrocolombiana se presenta mayor incidencia de dengue en la población no afrocolombiana. Aquí, los autores no indicaron datos o mediciones de esta condición para identificar si efectivamente las personas afrodescendientes se encontraban infectadas en el sitio de evaluación y si desarrollaron o no la enfermedad por lo que dichas conclusiones debían haber sido evaluadas con mayor rigurosidad antes de realizar afirmaciones de tal magnitud. 

A pesar de que existen características propias de los afrodescendientes que les pueden conferir una menor susceptibilidad a las manifestaciones graves del dengue, se sabe que la patogénesis por esta enfermedad es de carácter multifactorial, en la cual influyen variables como factores ambientales ^2^, la edad ^3^, el género ^4^, los diferentes tipos sanguíneos AB versus los tipos A, O y B ^5^, los polimorfismos de los alelos de los antígenos de leucocitos humanos^6^, y los factores asociados a la fuga vascular, entre otros. 

Las condiciones relacionadas a la genética del hospedero y a los factores ambientales podrían predisponer a las formas graves de la enfermedad, lo importante es no olvidar que la mayoría de ellos han sido resultado de conclusiones obtenidas por estudios de asociación. Inclusive, estudios recientes de meta-análisis sobre factores que pueden influir en las formas del dengue indican que la desnutrición tiene una asociación inversa con el síndrome de shock por dengue y la fiebre de dengue hemorrágica ^7^^,^^8^. Podríamos entonces, paradójicamente pensar que la desnutrición ofrece un estado de protección frente al desarrollo de la forma grave de la enfermedad, por lo tanto, ser de raza negra no significa estar protegido contra las formas del dengue, ni indica ser un factor de riesgo para otras etnias ^9^.

También es posible que el artículo este incurriendo en errores de medición o sesgo de información tipo mala-clasificación de acuerdo al auto reconocimiento de la raza de cada paciente ^10^. Una clasificación dicotómica como afro-colombiano o no afro-colombiano puede generar errores de medición, teniendo en cuenta que un paciente con antecedentes afros (mulato, zambo, mestizo, entre otros) puede clasificarse como no afro colombiano, sin embargo, puede presentar condiciones genéticas de sus antecesores, y de esta forma, vulnerabilidad ante posibles complicaciones de eventos infecciosos de origen externo. Es posible que de los 402/431 de casos severos de dengue con complicaciones en no afrocolombianos reportados por los autores, varios de estos, tengan elementos genéticos y rasgos de población afrocolombiana, lo cual generaría un error de medición por lo que resultaba necesario el realizar mediciones genéticas y biológicas que no fueron consideradas.

Por otro lado, llama la atención que al comparar las conclusiones obtenidas por Rojas et al. ^1^, con relación a los reportes epidemiológicos realizados por el Instituto Nacional de Salud (INS) en 2013, los mismos, distan sustancialmente entre sí, por ejemplo, en el informe del INS se notificó que los principales departamentos en orden de magnitud donde se presentaron casos por dengue clásico y dengue grave fueron Tolima, Valle, Santander, Norte de Santander y Cundinamarca; todos estos a excepción de Valle presentan baja población con raza negra ^11^.Aunque los autores manifiestan que no hay riesgo de una posible falacia ecológica, es claro que la gran mayoría de conclusiones se obtuvieron con análisis de datos o registros a nivel poblacional que exceden las conclusiones a nivel individual ^10^. Para evitar este tipo de situaciones se requieren validaciones con métodos multinivel o multi-etapas que permitan garantizar la congruencia entre los dos niveles de medición.Finalmente, cabe recordar que el dengue es una patología compleja cuyos mecanismos de las formas graves de la infección no han sido completamente elucidados por lo que se requieren estudios robustos y éticos para el establecimiento de nuevas teorías acerca de la patogenia viral, teniendo siempre una posición que evite atentar contra los derechos de los individuos de estudio.
