# Supplementary material for: Reflections on Afro-descendant origin and the outcome of dengue fever cases in Colombia
Source: Colomb Med (Cali). 2017 Jun 30;48(2):98–100. (PMC5625562)
Supplement: Related texts

# Reply: Authors Response: Reflections on Afro-descendant origin and the outcome of dengue fever cases in Colombia

**Authors:** Jorge Humberto Rojas Palacios, Alberto Alzate, Héctor Jairo Martínez Romero, Alberto Ignacio Concha Eastman

**Affiliations:** 1 Grupo de Epidemiología y Salud Pública, Secretaría de Salud Pública Municipal de Cali, Alcaldía Municipal de Santiago de Cali, Cali Colombia; 2 Grupo de Investigación en modelos y métodos matemáticos para el control y vigilancia del dengue, Cali, Colombia; 3 Grupo de Investigación en Epidemiología y Servicios (GRIEPIS). Postgrado de Epidemiología, Universidad Libre, Cali, Colombia; 4 Departamento de Matemáticas, Universidad del Valle. Cali, Colombia; 5 Alcaldía Municipal de Santiago de Cali, Cali, Colombia

Answer letter to the Editor

We have carefully read Rengifo et al.^1^, views on our research on dengue and ethnicity ^2^. Most of their observations point to the classification of ethnicity in the study design, in which we recognize a great difficulty to establish the category of Afro-descendant to each one of the individuals. Some proposals to classify that ethnic group in Latin American populations, based on genetic and biological tests, have shown a great miscegenation, which makes classification difficult ^3^ The method of self-recognition of the ethnicity is imperfect as we see in the methodology of our study; but it is still valid for the scientific community at this time, while other more efficient, reliable and robust methods arise ^4^ ^,^ ^5^.

We disagree with the letter correspondents in considering xenophobic any element of this research. The study design considered any risk of discrimination on the grounds that it directly affected the outcome, and that it safeguarded the principle of equity in the selection and evaluation of variables. Significant results were verified by adjusted models that in some cases refuted the original findings (cumulative incidence by age and ethnic origin). Our interpretations of the results also warn of a silent phase of dissemination of the epidemic in communities with a larger Afro-Colombian population, which may require sanitary adjustments to modify the case numbers of dengue and severe dengue.

Finally, we agree on the complexity of the disease and the limitations of ecological studies; but these two arguments do not detract from the value of the conclusions of the study beyond the limitations we recognize in the discussion. Public health research can contribute to better policy decisions in a region by allowing decision-makers to better understand a problem or situation. But defining what decisions authorities should take in health is a responsibility only of decision-makers, in which they have to take into account other aspects, such as social, economic, technical and also reliance on other sciences ^6^. In this way, our publication does not encompass defining moral and politically convenient decisions.

## References

[1] Rengifo AC, Durán MA, Ortíz Y, Rodriguez JM, Ospina ML (2017). Reflections on Afro-descendant origin and the outcome of dengue fever cases in Colombia. Colomb Med (Cali).

[2] Rojas-Palacios JH, Alzate A, Martinez-Romero HJ, Concha-Eastman AI (2016). Afro-Colombian ethnicity, a paradoxical protectiva factor against Dengue. Colomb Med (Cali).

[3] Wang S, Ray N, Rojas W, Parra MV, Bedoya G, Gallo C (2008). Geographic patterns of genome admixture in Latin American mestizos. PLoS Genet.

[4] Agudelo-Suárez AA, Martínez-Herrera E, Posada-López A, Rocha-Buelvas A (2016). Ethnicity and health in Colombia what do self-perceived health indicators tell us?. Ethn Dis.

[5] Larifla L, Beaney KE, Foucan L, Bangou J, Michel CT, Martino J (2016). Influence of genetic risk factors on coronary heart disease occurrence in Afro-Caribbeans. Can J Cardiol.

[6] Feldhay R (2013). The fragile boundary between the political and the academic. Israel Studies Review.

# Reply: Respuesta: Reflecciones sobre el origen Afro-descendiente y el resultado de casos de dengue en Colombia

Respuesta carta al Editor

Hemos leído con atención las apreciaciones de Rengifo *et al*.^1^, respecto a nuestra investigación sobre dengue y etnia ^2^. La mayoría de sus observaciones apuntan hacia la clasificación de etnia en el diseño del estudio; donde reconocemos una gran dificultad para establecer la categoría de afrodescendiente a cada uno de los individuos. Algunas propuestas para clasificar la etnia en poblaciones latinoamericanas con base en pruebas genéticas y biológicas han mostrado un gran mestizaje que dificulta la clasificación ^3^. El método de la auto reconocimiento de la etnia es imperfecto como lo advertimos en la metodología de nuestro estudio; pero válido para la comunidad científica en este momento, mientras se propongan otros métodos eficientes, más confiables y robustos ^4^ ^,^ ^5^. 

Diferimos con los corresponsales de la carta en considerar xenofóbico cualquier elemento de la investigación. En e l diseño del estudio consideró cualquier riesgo de discriminación por considerar que afectaba directamente el resultado y se resguardo el principio de equidad en la selección y evaluación de las variables. Los resultados significativos fueron comprobados por modelos ajustados que en algunos casos refutaron los hallazgos originales (incidencia acumulada por edad y origen étnico). Nuestras interpretaciones de los resultados también alertan de una fase silenciosa de diseminación de la epidemia en comunas con mayor población afrocolombiana que puede requerir ajustes sanitarios para modificar las cifras de caso de dengue y dengue grave. 

Por último, coincidimos en la complejidad de la enfermedad y las limitaciones de los estudios ecológicos; pero, estos dos argumentos no restan el valor de las conclusiones del estudio mas alla de las limitaciones que reconocemos en la discusión. Las investigaciones en salud pública pueden contribuir a que se tomen mejores decisiones políticas en una región, porque le permite a los gobernantes entender mejor un problema o una situación. Pero, definir que decisiones debe tomar una autoridad en salud es una responsabilidad únicamente del gobernante, donde tiene que tener en cuenta otros aspectos, como los sociales, económicos, técnicos y además apoyarse en otras ciencias ^6^. De este modo, nuestra publicación no alcanza a definir sobre las decisiones morales y políticamente convenientes.
